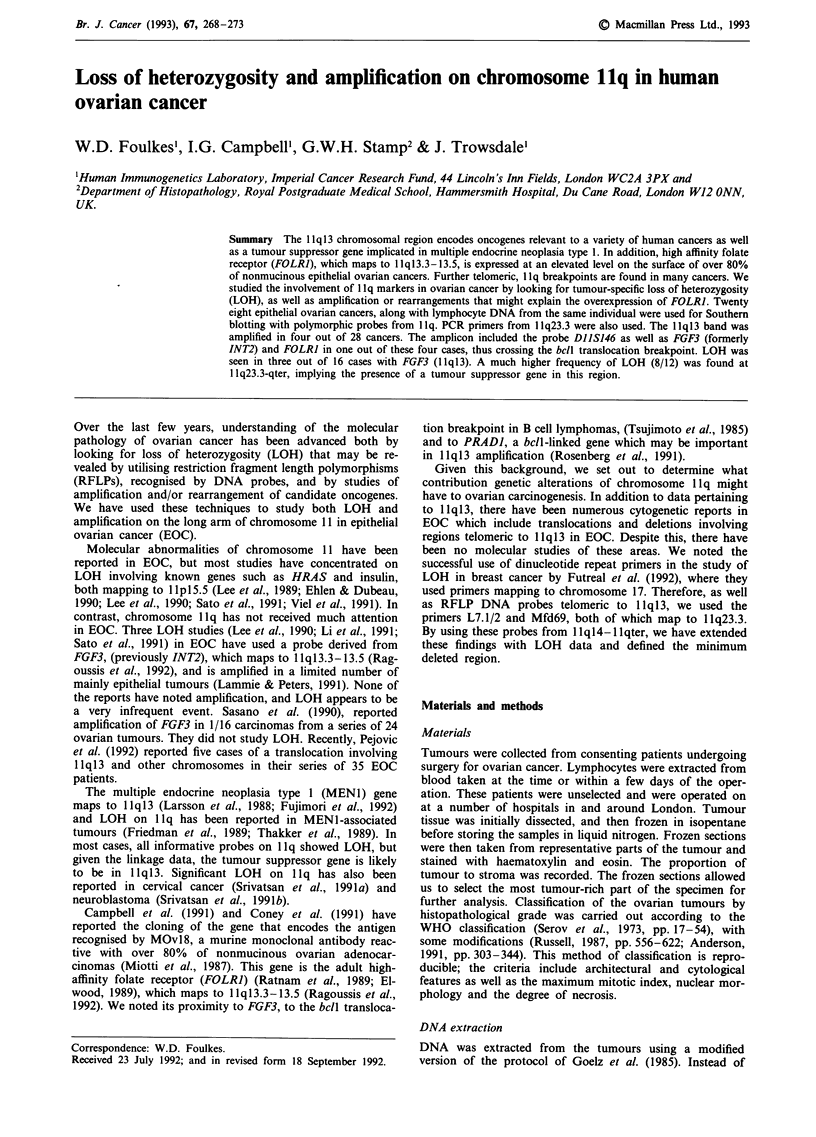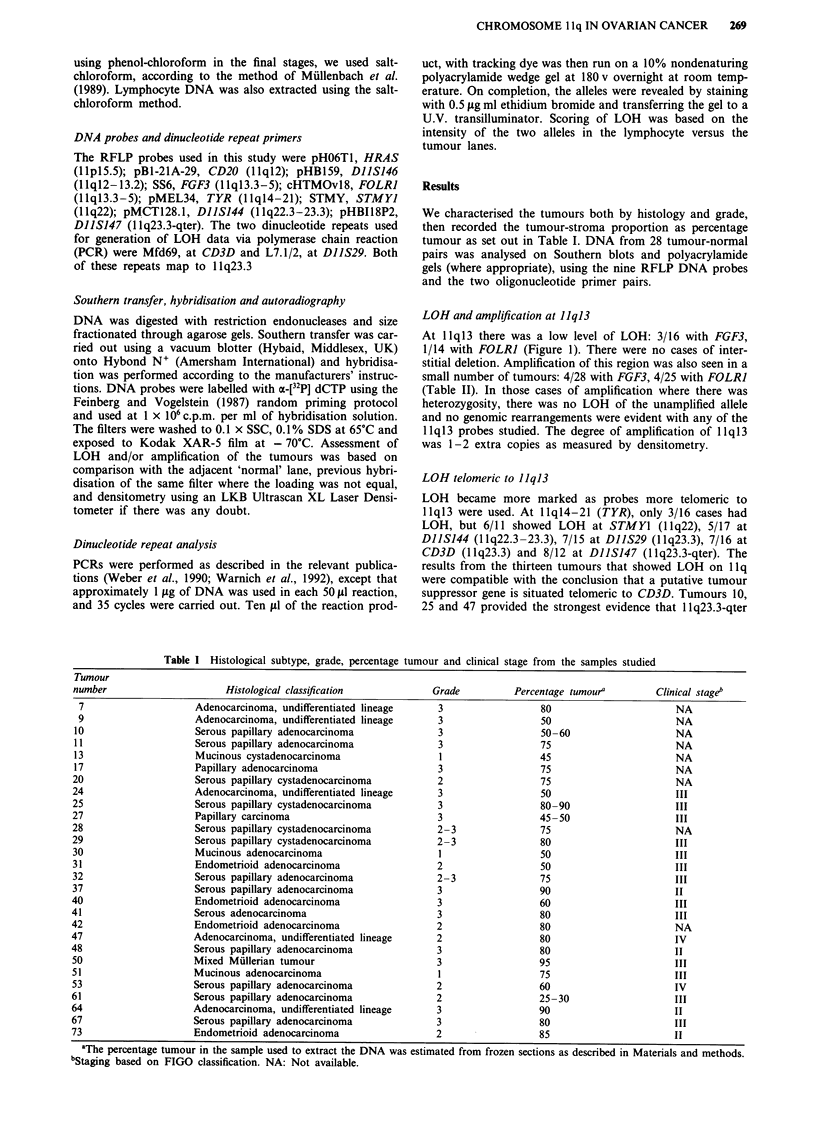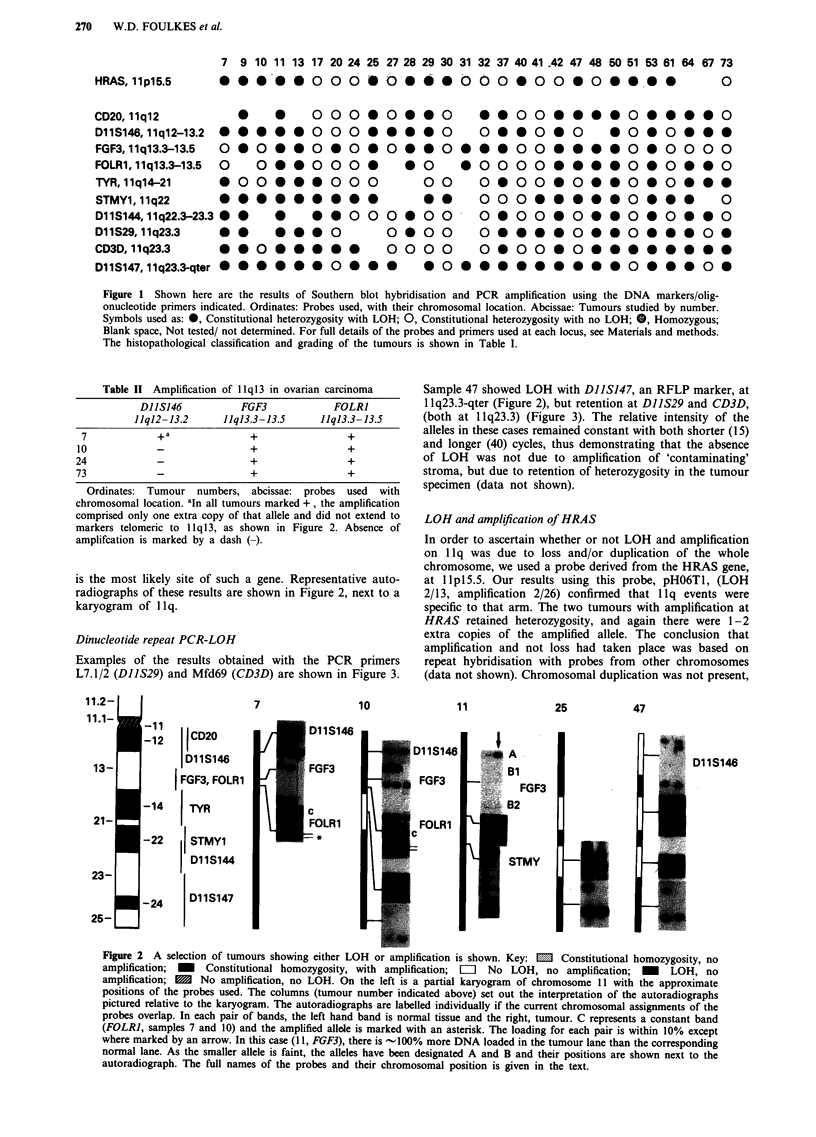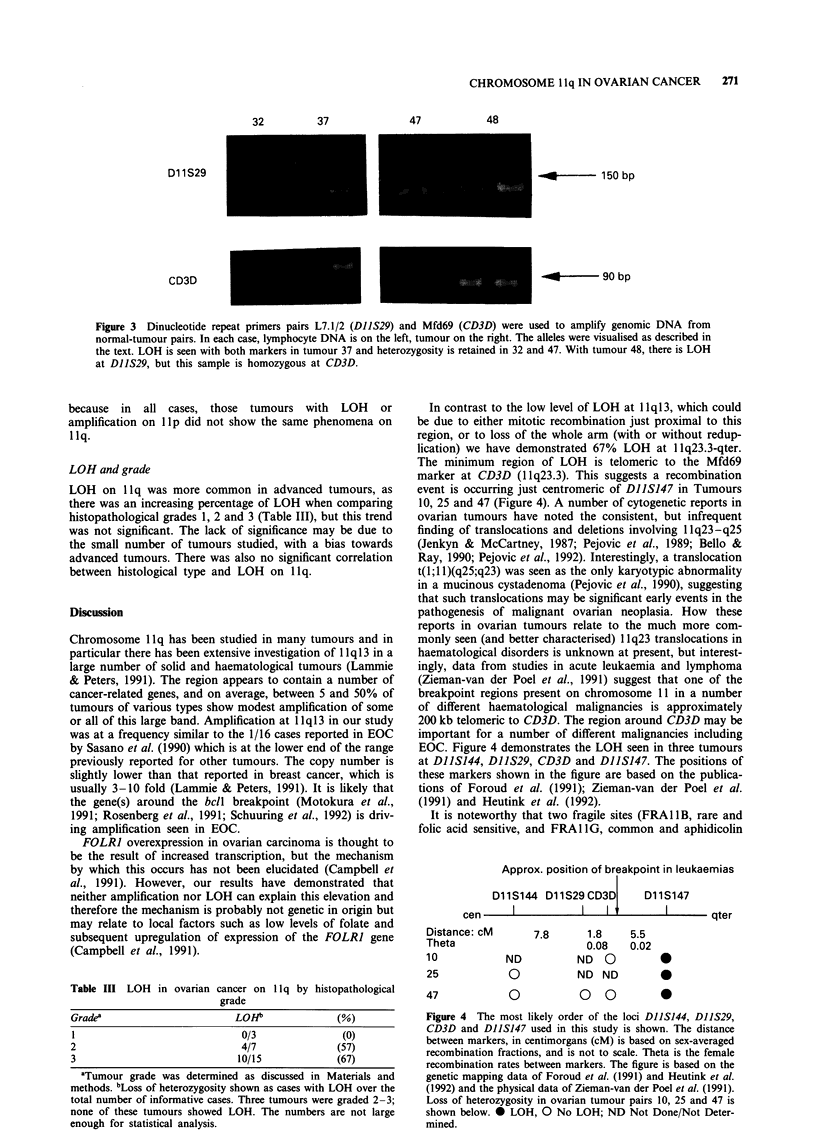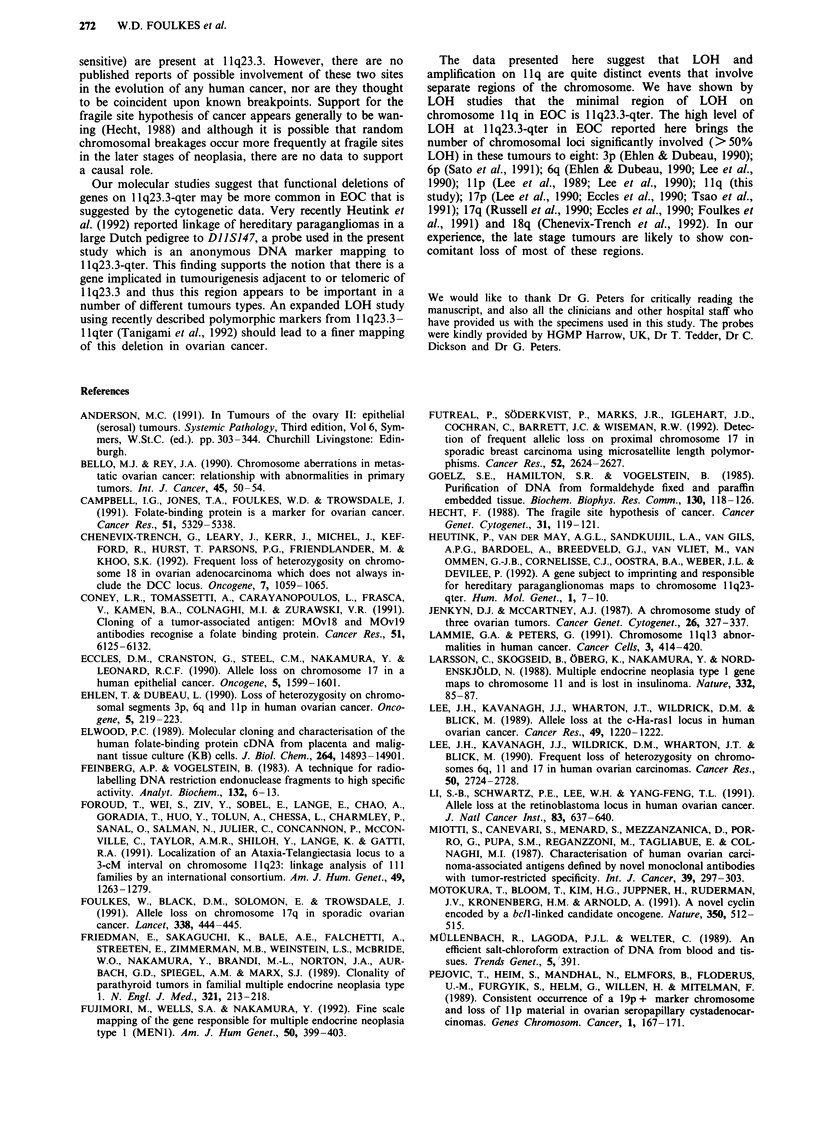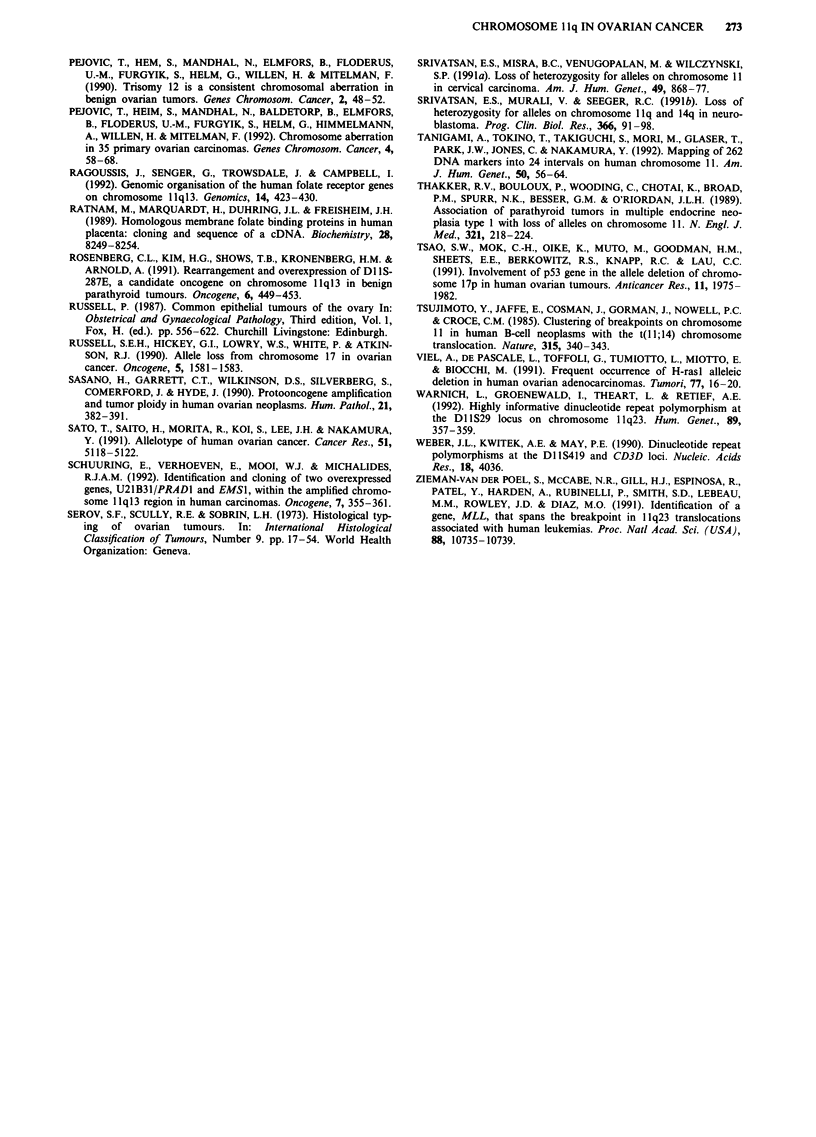# Loss of heterozygosity and amplification on chromosome 11q in human ovarian cancer.

**DOI:** 10.1038/bjc.1993.51

**Published:** 1993-02

**Authors:** W. D. Foulkes, I. G. Campbell, G. W. Stamp, J. Trowsdale

**Affiliations:** Human Immunogenetics Laboratory, Imperial Cancer Research Fund, London, UK.

## Abstract

**Images:**


Br. J. Cancer (1993), 67, 268-273 © Macmillan Press Ltd., 1993

### Loss of heterozygosity and amplification on chromosome 11q in human ovarian cancer

W.D. Foulkes[1], I.G. Campbell[1], G.W.H. Stamp[2] & J. Trowsdale[1]

[1]*Human Immunogenetics Laboratory, Imperial Cancer Research Fund, 44 Lincoln's Inn Fields, London WC2A 3PX and* [2]*Department of Histopathology, Royal Postgraduate Medical School, Hammersmith Hospital, Du Cane Road, London W12 0NN, UK.*

**Summary** The 11q13 chromosomal region encodes oncogenes relevant to a variety of human cancers as well as a tumour suppressor gene implicated in multiple endocrine neoplasia type 1. In addition, high affinity folate receptor (*FOLR1*), which maps to 11q13.3-13.5, is expressed at an elevated level on the surface of over 80% of nonmucinous epithelial ovarian cancers. Further telomeric, 11q breakpoints are found in many cancers. We studied the involvement of 11q markers in ovarian cancer by looking for tumour-specific loss of heterozygosity (LOH), as well as amplification or rearrangements that might explain the overexpression of *FOLR1*. Twenty eight epithelial ovarian cancers, along with lymphocyte DNA from the same individual were used for Southern blotting with polymorphic probes from 11q. PCR primers from 11q23.3 were also used. The 11q13 band was amplified in four out of 28 cancers. The amplicon included the probe *D11S146* as well as *FGF3* (formerly *INT2*) and *FOLR1* in one out of these four cases, thus crossing the *bcl*1 translocation breakpoint. LOH was seen in three out of 16 cases with *FGF3* (11q13). A much higher frequency of LOH (8/12) was found at 11q23.3-qter, implying the presence of a tumour suppressor gene in this region.

Over the last few years, understanding of the molecular pathology of ovarian cancer has been advanced both by looking for loss of heterozygosity (LOH) that may be revealed by utilising restriction fragment length polymorphisms (RFLPs), recognised by DNA probes, and by studies of amplification and/or rearrangement of candidate oncogenes. We have used these techniques to study both LOH and amplification on the long arm of chromosome 11 in epithelial ovarian cancer (EOC).

Molecular abnormalities of chromosome 11 have been reported in EOC, but most studies have concentrated on LOH involving known genes such as *HRAS* and insulin, both mapping to 11p15.5 (Lee *et al.*, 1989; Ehlen & Dubeau, 1990; Lee *et al.*, 1990; Sato *et al.*, 1991; Viel *et al.*, 1991). In contrast, chromosome 11q has not received much attention in EOC. Three LOH studies (Lee *et al.*, 1990; Li *et al.*, 1991; Sato *et al.*, 1991) in EOC have used a probe derived from *FGF3*, (previously *INT2*), which maps to 11q13.3-13.5 (Ragoussis *et al.*, 1992), and is amplified in a limited number of mainly epithelial tumours (Lammie & Peters, 1991). None of the reports have noted amplification, and LOH appears to be a very infrequent event. Sasano *et al.* (1990), reported amplification of *FGF3* in 1/16 carcinomas from a series of 24 ovarian tumours. They did not study LOH. Recently, Pejovic *et al.* (1992) reported five cases of a translocation involving 11q13 and other chromosomes in their series of 35 EOC patients.

The multiple endocrine neoplasia type 1 (MEN1) gene maps to 11q13 (Larsson *et al.*, 1988; Fujimori *et al.*, 1992) and LOH on 11q has been reported in MEN1-associated tumours (Friedman *et al.*, 1989; Thakker *et al.*, 1989). In most cases, all informative probes on 11q showed LOH, but given the linkage data, the tumour suppressor gene is likely to be in 11q13. Significant LOH on 11q has also been reported in cervical cancer (Srivatsan *et al.*, 1991*a*) and neuroblastoma (Srivatsan *et al.*, 1991*b*).

Campbell *et al.* (1991) and Coney *et al.* (1991) have reported the cloning of the gene that encodes the antigen recognised by MOv18, a murine monoclonal antibody reactive with over 80% of nonmucinous ovarian adenocarcinomas (Miotti *et al.*, 1987). This gene is the adult high-affinity folate receptor (*FOLR1*) (Ratnam *et al.*, 1989; Elwood, 1989), which maps to 11q13.3-13.5 (Ragoussis *et al.*, 1992). We noted its proximity to *FGF3*, to the *bcl*1 translocation breakpoint in B cell lymphomas, (Tsujimoto *et al.*, 1985) and to *PRAD1*, a *bcl*1-linked gene which may be important in 11q13 amplification (Rosenberg *et al.*, 1991).

Given this background, we set out to determine what contribution genetic alterations of chromosome 11q might have to ovarian carcinogenesis. In addition to data pertaining to 11q13, there have been numerous cytogenetic reports in EOC which include translocations and deletions involving regions telomeric to 11q13 in EOC. Despite this, there have been no molecular studies of these areas. We noted the successful use of dinucleotide repeat primers in the study of LOH in breast cancer by Futreal *et al.* (1992), where they used primers mapping to chromosome 17. Therefore, as well as RFLP DNA probes telomeric to 11q13, we used the primers L7.1/2 and Mfd69, both of which map to 11q23.3. By using these probes from 11q14-11qter, we have extended these findings with LOH data and defined the minimum deleted region.

### Materials and methods

### *Materials*

Tumours were collected from consenting patients undergoing surgery for ovarian cancer. Lymphocytes were extracted from blood taken at the time or within a few days of the operation. These patients were unselected and were operated on at a number of hospitals in and around London. Tumour tissue was initially dissected, and then frozen in isopentane before storing the samples in liquid nitrogen. Frozen sections were then taken from representative parts of the tumour and stained with haematoxylin and eosin. The proportion of tumour to stroma was recorded. The frozen sections allowed us to select the most tumour-rich part of the specimen for further analysis. Classification of the ovarian tumours by histopathological grade was carried out according to the WHO classification (Serov *et al.*, 1973, pp. 17-54), with some modifications (Russell, 1987, pp. 556-622; Anderson, 1991, pp. 303-344). This method of classification is reproducible; the criteria include architectural and cytological features as well as the maximum mitotic index, nuclear morphology and the degree of necrosis.

### *DNA extraction*

DNA was extracted from the tumours using a modified version of the protocol of Goelz *et al.* (1985). Instead of

Correspondence: W.D. Foulkes.
Received 23 July 1992; and in revised form 18 September 1992.

using phenol-chloroform in the final stages, we used salt-chloroform, according to the method of Müllenbach *et al.* (1989). Lymphocyte DNA was also extracted using the salt-chloroform method.

### *DNA probes and dinucleotide repeat primers*

The RFLP probes used in this study were pH06T1, *HRAS* (11p15.5); pB1-21A-29, *CD20* (11q12); pHB159, *D11S146* (11q12-13.2); SS6, *FGF3* (11q13.3-5); cHTMOv18, *FOLR1* (11q13.3-5); pMEL34, *TYR* (11q14-21); STMY, *STMY1* (11q22); pMCT128.1, *D11S144* (11q22.3-23.3); pHBI18P2, *D11S147* (11q23.3-qter). The two dinucleotide repeats used for generation of LOH data via polymerase chain reaction (PCR) were Mfd69, at *CD3D* and L7.1/2, at *D11S29*. Both of these repeats map to 11q23.3

### *Southern transfer, hybridisation and autoradiography*

DNA was digested with restriction endonucleases and size fractionated through agarose gels. Southern transfer was carried out using a vacuum blotter (Hybaid, Middlesex, UK) onto Hybond $N^+$ (Amersham International) and hybridisation was performed according to the manufacturers' instructions. DNA probes were labelled with $\alpha$-[$^{32}$P] dCTP using the Feinberg and Vogelstein (1987) random priming protocol and used at $1 \times 10^6$ c.p.m. per ml of hybridisation solution. The filters were washed to 0.1 × SSC, 0.1% SDS at 65°C and exposed to Kodak XAR-5 film at − 70°C. Assessment of LOH and/or amplification of the tumours was based on comparison with the adjacent 'normal' lane, previous hybridisation of the same filter where the loading was not equal, and densitometry using an LKB Ultrascan XL Laser Densitometer if there was any doubt.

### *Dinucleotide repeat analysis*

PCRs were performed as described in the relevant publications (Weber *et al.*, 1990; Warnich *et al.*, 1992), except that approximately 1 µg of DNA was used in each 50 µl reaction, and 35 cycles were carried out. Ten µl of the reaction product, with tracking dye was then run on a 10% nondenaturing polyacrylamide wedge gel at 180 v overnight at room temperature. On completion, the alleles were revealed by staining with 0.5 µg ml ethidium bromide and transferring the gel to a U.V. transilluminator. Scoring of LOH was based on the intensity of the two alleles in the lymphocyte versus the tumour lanes.

### Results

We characterised the tumours both by histology and grade, then recorded the tumour-stroma proportion as percentage tumour as set out in Table I. DNA from 28 tumour-normal pairs was analysed on Southern blots and polyacrylamide gels (where appropriate), using the nine RFLP DNA probes and the two oligonucleotide primer pairs.

### *LOH and amplification at 11q13*

At 11q13 there was a low level of LOH: 3/16 with *FGF3*, 1/14 with *FOLR1* (Figure 1). There were no cases of interstitial deletion. Amplification of this region was also seen in a small number of tumours: 4/28 with *FGF3*, 4/25 with *FOLR1* (Table II). In those cases of amplification where there was heterozygosity, there was no LOH of the unamplified allele and no genomic rearrangements were evident with any of the 11q13 probes studied. The degree of amplification of 11q13 was 1-2 extra copies as measured by densitometry.

### *LOH telomeric to 11q13*

LOH became more marked as probes more telomeric to 11q13 were used. At 11q14-21 (*TYR*), only 3/16 cases had LOH, but 6/11 showed LOH at *STMY*1 (11q22), 5/17 at *D11S144* (11q22.3-23.3), 7/15 at *D11S29* (11q23.3), 7/16 at *CD3D* (11q23.3) and 8/12 at *D11S147* (11q23.3-qter). The results from the thirteen tumours that showed LOH on 11q were compatible with the conclusion that a putative tumour suppressor gene is situated telomeric to *CD3D*. Tumours 10, 25 and 47 provided the strongest evidence that 11q23.3-qter

**Table I** Histological subtype, grade, percentage tumour and clinical stage from the samples studied

| *Tumour number* | *Histological classification* | *Grade* | *Percentage tumour*[a] | *Clinical stage*[b] |
|---|---|---|---|---|
| 7 | Adenocarcinoma, undifferentiated lineage | 3 | 80 | NA |
| 9 | Adenocarcinoma, undifferentiated lineage | 3 | 50 | NA |
| 10 | Serous papillary adenocarcinoma | 3 | 50-60 | NA |
| 11 | Serous papillary adenocarcinoma | 3 | 75 | NA |
| 13 | Mucinous cystadenocarcinoma | 1 | 45 | NA |
| 17 | Papillary adenocarcinoma | 3 | 75 | NA |
| 20 | Serous papillary cystadenocarcinoma | 2 | 75 | NA |
| 24 | Adenocarcinoma, undifferentiated lineage | 3 | 50 | III |
| 25 | Serous papillary cystadenocarcinoma | 3 | 80-90 | III |
| 27 | Papillary carcinoma | 3 | 45-50 | III |
| 28 | Serous papillary cystadenocarcinoma | 2-3 | 75 | NA |
| 29 | Serous papillary cystadenocarcinoma | 2-3 | 80 | III |
| 30 | Mucinous adenocarcinoma | 1 | 50 | III |
| 31 | Endometrioid adenocarcinoma | 2 | 50 | III |
| 32 | Serous papillary adenocarcinoma | 2-3 | 75 | III |
| 37 | Serous papillary adenocarcinoma | 3 | 90 | II |
| 40 | Endometrioid adenocarcinoma | 3 | 60 | III |
| 41 | Serous adenocarcinoma | 3 | 80 | III |
| 42 | Endometrioid adenocarcinoma | 2 | 80 | NA |
| 47 | Adenocarcinoma, undifferentiated lineage | 2 | 80 | IV |
| 48 | Serous papillary adenocarcinoma | 3 | 80 | II |
| 50 | Mixed Müllerian tumour | 3 | 95 | III |
| 51 | Mucinous adenocarcinoma | 1 | 75 | III |
| 53 | Serous papillary adenocarcinoma | 2 | 60 | IV |
| 61 | Serous papillary adenocarcinoma | 2 | 25-30 | III |
| 64 | Adenocarcinoma, undifferentiated lineage | 3 | 90 | II |
| 67 | Serous papillary adenocarcinoma | 3 | 80 | III |
| 73 | Endometrioid adenocarcinoma | 2 | 85 | II |

[a]The percentage tumour in the sample used to extract the DNA was estimated from frozen sections as described in Materials and methods. [b]Staging based on FIGO classification. NA: Not available.

**Figure 1** Shown here are the results of Southern blot hybridisation and PCR amplification using the DNA markers/oligonucleotide primers indicated. Ordinates: Probes used, with their chromosomal location. Abcissae: Tumours studied by number. Symbols used as: ●, Constitutional heterozygosity with LOH; ○, Constitutional heterozygosity with no LOH; ◍, Homozygous; Blank space, Not tested/ not determined. For full details of the probes and primers used at each locus, see Materials and methods. The histopathological classification and grading of the tumours is shown in Table I.

**Table II** Amplification of 11q13 in ovarian carcinoma

| | *D11S146* *11q12–13.2* | *FGF3* *11q13.3–13.5* | *FOLR1* *11q13.3–13.5* |
|---|---|---|---|
| 7 | +[a] | + | + |
| 10 | – | + | + |
| 24 | – | + | + |
| 73 | – | + | + |

Ordinates: Tumour numbers, abcissae: probes used with chromosomal location. [a]In all tumours marked +, the amplification comprised only one extra copy of that allele and did not extend to markers telomeric to 11q13, as shown in Figure 2. Absence of amplifcation is marked by a dash (–).

is the most likely site of such a gene. Representative autoradiographs of these results are shown in Figure 2, next to a karyogram of 11q.

### *Dinucleotide repeat PCR-LOH*

Examples of the results obtained with the PCR primers L7.1/2 (*D11S29*) and Mfd69 (*CD3D*) are shown in Figure 3. Sample 47 showed LOH with *D11S147*, an RFLP marker, at 11q23.3-qter (Figure 2), but retention at *D11S29* and *CD3D*, (both at 11q23.3) (Figure 3). The relative intensity of the alleles in these cases remained constant with both shorter (15) and longer (40) cycles, thus demonstrating that the absence of LOH was not due to amplification of 'contaminating' stroma, but due to retention of heterozygosity in the tumour specimen (data not shown).

### *LOH and amplification of HRAS*

In order to ascertain whether or not LOH and amplification on 11q was due to loss and/or duplication of the whole chromosome, we used a probe derived from the HRAS gene, at 11p15.5. Our results using this probe, pH06T1, (LOH 2/13, amplification 2/26) confirmed that 11q events were specific to that arm. The two tumours with amplification at *HRAS* retained heterozygosity, and again there were 1–2 extra copies of the amplified allele. The conclusion that amplification and not loss had taken place was based on repeat hybridisation with probes from other chromosomes (data not shown). Chromosomal duplication was not present,

**Figure 2** A selection of tumours showing either LOH or amplification is shown. Key: ▒ Constitutional homozygosity, no amplification; ■ Constitutional homozygosity, with amplification; □ No LOH, no amplification; ■ LOH, no amplification; ▨ No amplification, no LOH. On the left is a partial karyogram of chromosome 11 with the approximate positions of the probes used. The columns (tumour number indicated above) set out the interpretation of the autoradiographs pictured relative to the karyogram. The autoradiographs are labelled individually if the current chromosomal assignments of the probes overlap. In each pair of bands, the left hand band is normal tissue and the right, tumour. C represents a constant band (*FOLR1*, samples 7 and 10) and the amplified allele is marked with an asterisk. The loading for each pair is within 10% except where marked by an arrow. In this case (11, *FGF3*), there is ~100% more DNA loaded in the tumour lane than the corresponding normal lane. As the smaller allele is faint, the alleles have been designated A and B and their positions are shown next to the autoradiograph. The full names of the probes and their chromosomal position is given in the text.

**Figure 3** Dinucleotide repeat primers pairs L7.1/2 (*D11S29*) and Mfd69 (*CD3D*) were used to amplify genomic DNA from normal-tumour pairs. In each case, lymphocyte DNA is on the left, tumour on the right. The alleles were visualised as described in the text. LOH is seen with both markers in tumour 37 and heterozygosity is retained in 32 and 47. With tumour 48, there is LOH at *D11S29*, but this sample is homozygous at *CD3D*.

because in all cases, those tumours with LOH or amplification on 11p did not show the same phenomena on 11q.

### *LOH and grade*

LOH on 11q was more common in advanced tumours, as there was an increasing percentage of LOH when comparing histopathological grades 1, 2 and 3 (Table III), but this trend was not significant. The lack of significance may be due to the small number of tumours studied, with a bias towards advanced tumours. There was also no significant correlation between histological type and LOH on 11q.

### Discussion

Chromosome 11q has been studied in many tumours and in particular there has been extensive investigation of 11q13 in a large number of solid and haematological tumours (Lammie & Peters, 1991). The region appears to contain a number of cancer-related genes, and on average, between 5 and 50% of tumours of various types show modest amplification of some or all of this large band. Amplification at 11q13 in our study was at a frequency similar to the 1/16 cases reported in EOC by Sasano *et al.* (1990) which is at the lower end of the range previously reported for other tumours. The copy number is slightly lower than that reported in breast cancer, which is usually 3–10 fold (Lammie & Peters, 1991). It is likely that the gene(s) around the *bcl*1 breakpoint (Motokura *et al.*, 1991; Rosenberg *et al.*, 1991; Schuuring *et al.*, 1992) is driving amplification seen in EOC.

*FOLR1* overexpression in ovarian carcinoma is thought to be the result of increased transcription, but the mechanism by which this occurs has not been elucidated (Campbell *et al.*, 1991). However, our results have demonstrated that neither amplification nor LOH can explain this elevation and therefore the mechanism is probably not genetic in origin but may relate to local factors such as low levels of folate and subsequent upregulation of expression of the *FOLR1* gene (Campbell *et al.*, 1991).

**Table III** LOH in ovarian cancer on 11q by histopathological grade

| *Grade*[a] | *LOH*[b] | (%) |
|---|---|---|
| 1 | 0/3 | (0) |
| 2 | 4/7 | (57) |
| 3 | 10/15 | (67) |

[a]Tumour grade was determined as discussed in Materials and methods. [b]Loss of heterozygosity shown as cases with LOH over the total number of informative cases. Three tumours were graded 2–3; none of these tumours showed LOH. The numbers are not large enough for statistical analysis.

In contrast to the low level of LOH at 11q13, which could be due to either mitotic recombination just proximal to this region, or to loss of the whole arm (with or without reduplication) we have demonstrated 67% LOH at 11q23.3-qter. The minimum region of LOH is telomeric to the Mfd69 marker at *CD3D* (11q23.3). This suggests a recombination event is occurring just centromeric of *D11S147* in Tumours 10, 25 and 47 (Figure 4). A number of cytogenetic reports in ovarian tumours have noted the consistent, but infrequent finding of translocations and deletions involving 11q23–q25 (Jenkyn & McCartney, 1987; Pejovic *et al.*, 1989; Bello & Ray, 1990; Pejovic *et al.*, 1992). Interestingly, a translocation t(1;11)(q25;q23) was seen as the only karyotypic abnormality in a mucinous cystadenoma (Pejovic *et al.*, 1990), suggesting that such translocations may be significant early events in the pathogenesis of malignant ovarian neoplasia. How these reports in ovarian tumours relate to the much more commonly seen (and better characterised) 11q23 translocations in haematological disorders is unknown at present, but interestingly, data from studies in acute leukaemia and lymphoma (Zieman-van der Poel *et al.*, 1991) suggest that one of the breakpoint regions present on chromosome 11 in a number of different haematological malignancies is approximately 200 kb telomeric to *CD3D*. The region around *CD3D* may be important for a number of different malignancies including EOC. Figure 4 demonstrates the LOH seen in three tumours at *D11S144*, *D11S29*, *CD3D* and *D11S147*. The positions of these markers shown in the figure are based on the publications of Foroud *et al.* (1991); Zieman-van der Poel *et al.* (1991) and Heutink *et al.* (1992).

It is noteworthy that two fragile sites (FRA11B, rare and folic acid sensitive, and FRA11G, common and aphidicolin

**Figure 4** The most likely order of the loci *D11S144*, *D11S29*, *CD3D* and *D11S147* used in this study is shown. The distance between markers, in centimorgans (cM) is based on sex-averaged recombination fractions, and is not to scale. Theta is the female recombination rates between markers. The figure is based on the genetic mapping data of Foroud *et al.* (1991) and Heutink *et al.* (1992) and the physical data of Zieman-van der Poel *et al.* (1991). Loss of heterozygosity in ovarian tumour pairs 10, 25 and 47 is shown below. ● LOH, ○ No LOH; ND Not Done/Not Determined.

sensitive) are present at 11q23.3. However, there are no published reports of possible involvement of these two sites in the evolution of any human cancer, nor are they thought to be coincident upon known breakpoints. Support for the fragile site hypothesis of cancer appears generally to be waning (Hecht, 1988) and although it is possible that random chromosomal breakages occur more frequently at fragile sites in the later stages of neoplasia, there are no data to support a causal role.

Our molecular studies suggest that functional deletions of genes on 11q23.3-qter may be more common in EOC that is suggested by the cytogenetic data. Very recently Heutink et al. (1992) reported linkage of hereditary paragangliomas in a large Dutch pedigree to *D11S147*, a probe used in the present study which is an anonymous DNA marker mapping to 11q23.3-qter. This finding supports the notion that there is a gene implicated in tumourigenesis adjacent to or telomeric of 11q23.3 and thus this region appears to be important in a number of different tumours types. An expanded LOH study using recently described polymorphic markers from 11q23.3–11qter (Tanigami et al., 1992) should lead to a finer mapping of this deletion in ovarian cancer.

The data presented here suggest that LOH and amplification on 11q are quite distinct events that involve separate regions of the chromosome. We have shown by LOH studies that the minimal region of LOH on chromosome 11q in EOC is 11q23.3-qter. The high level of LOH at 11q23.3-qter in EOC reported here brings the number of chromosomal loci significantly involved (>50% LOH) in these tumours to eight: 3p (Ehlen & Dubeau, 1990); 6p (Sato et al., 1991); 6q (Ehlen & Dubeau, 1990; Lee et al., 1990); 11p (Lee et al., 1989; Lee et al., 1990); 11q (this study); 17p (Lee et al., 1990; Eccles et al., 1990; Tsao et al., 1991); 17q (Russell et al., 1990; Eccles et al., 1990; Foulkes et al., 1991) and 18q (Chenevix-Trench et al., 1992). In our experience, the late stage tumours are likely to show concomitant loss of most of these regions.

We would like to thank Dr G. Peters for critically reading the manuscript, and also all the clinicians and other hospital staff who have provided us with the specimens used in this study. The probes were kindly provided by HGMP Harrow, UK, Dr T. Tedder, Dr C. Dickson and Dr G. Peters.

### References

ANDERSON, M.C. (1991). In Tumours of the ovary II: epithelial (serosal) tumours. *Systemic Pathology*, Third edition, Vol 6, Symmers, W.St.C. (ed.). pp. 303–344. Churchill Livingstone: Edinburgh.

BELLO, M.J. & REY, J.A. (1990). Chromosome aberrations in metastatic ovarian cancer: relationship with abnormalities in primary tumors. *Int. J. Cancer*, **45**, 50–54.

CAMPBELL, I.G., JONES, T.A., FOULKES, W.D. & TROWSDALE, J. (1991). Folate-binding protein is a marker for ovarian cancer. *Cancer Res.*, **51**, 5329–5338.

CHENEVIX-TRENCH, G., LEARY, J., KERR, J., MICHEL, J., KEFFORD, R., HURST, T. PARSONS, P.G., FRIENDLANDER, M. & KHOO, S.K. (1992). Frequent loss of heterozygosity on chromosome 18 in ovarian adenocarcinoma which does not always include the DCC locus. *Oncogene*, **7**, 1059–1065.

CONEY, L.R., TOMASSETTI, A., CARAYANOPOULOS, L., FRASCA, V., KAMEN, B.A., COLNAGHI, M.I. & ZURAWSKI, V.R. (1991). Cloning of a tumor-associated antigen: MOv18 and MOv19 antibodies recognise a folate binding protein. *Cancer Res.*, **51**, 6125–6132.

ECCLES, D.M., CRANSTON, G., STEEL, C.M., NAKAMURA, Y. & LEONARD, R.C.F. (1990). Allele loss on chromosome 17 in a human epithelial cancer. *Oncogene*, **5**, 1599–1601.

EHLEN, T. & DUBEAU, L. (1990). Loss of heterozygosity on chromosomal segments 3p, 6q and 11p in human ovarian cancer. *Oncogene*, **5**, 219–223.

ELWOOD, P.C. (1989). Molecular cloning and characterisation of the human folate-binding protein cDNA from placenta and malignant tissue culture (KB) cells. *J. Biol. Chem.*, **264**, 14893–14901.

FEINBERG, A.P. & VOGELSTEIN, B. (1983). A technique for radiolabelling DNA restriction endonuclease fragments to high specific activity. *Analyt. Biochem.*, **132**, 6–13.

FOROUD, T., WEI, S., ZIV, Y., SOBEL, E., LANGE, E., CHAO, A., GORADIA, T., HUO, Y., TOLUN, A., CHESSA, L., CHARMLEY, P., SANAL, O., SALMAN, N., JULIER, C., CONCANNON, P., MCCONVILLE, C., TAYLOR, A.M.R., SHILOH, Y., LANGE, K. & GATTI, R.A. (1991). Localization of an Ataxia-Telangiectasia locus to a 3-cM interval on chromosome 11q23: linkage analysis of 111 families by an international consortium. *Am. J. Hum. Genet.*, **49**, 1263–1279.

FOULKES, W., BLACK, D.M., SOLOMON, E. & TROWSDALE, J. (1991). Allele loss on chromosome 17q in sporadic ovarian cancer. *Lancet*, **338**, 444–445.

FRIEDMAN, E., SAKAGUCHI, K., BALE, A.E., FALCHETTI, A., STREETEN, E., ZIMMERMAN, M.B., WEINSTEIN, L.S., MCBRIDE, W.O., NAKAMURA, Y., BRANDI, M.-L., NORTON, J.A., AURBACH, G.D., SPIEGEL, A.M. & MARX, S.J. (1989). Clonality of parathyroid tumors in familial multiple endocrine neoplasia type 1. *N. Engl. J. Med.*, **321**, 213–218.

FUJIMORI, M., WELLS, S.A. & NAKAMURA, Y. (1992). Fine scale mapping of the gene responsible for multiple endocrine neoplasia type 1 (MEN1). *Am. J. Hum Genet.*, **50**, 399–403.

FUTREAL, P., SÖDERKVIST, P., MARKS, J.R., IGLEHART, J.D., COCHRAN, C., BARRETT, J.C. & WISEMAN, R.W. (1992). Detection of frequent allelic loss on proximal chromosome 17 in sporadic breast carcinoma using microsatellite length polymorphisms. *Cancer Res.*, **52**, 2624–2627.

GOELZ, S.E., HAMILTON, S.R. & VOGELSTEIN, B. (1985). Purification of DNA from formaldehyde fixed and paraffin embedded tissue. *Biochem. Biophys. Res. Comm.*, **130**, 118–126.

HECHT, F. (1988). The fragile site hypothesis of cancer. *Cancer Genet. Cytogenet.*, **31**, 119–121.

HEUTINK, P., VAN DER MAY, A.G.L., SANDKUIJIL, L.A., VAN GILS, A.P.G., BARDOEL, A., BREEDVELD, G.J., VAN VLIET, M., VAN OMMEN, G.-J.B., CORNELISSE, C.J., OOSTRA, B.A., WEBER, J.L. & DEVILEE, P. (1992). A gene subject to imprinting and responsible for hereditary paraganglionomas maps to chromosome 11q23-qter. *Hum. Mol. Genet.*, **1**, 7–10.

JENKYN, D.J. & MCCARTNEY, A.J. (1987). A chromosome study of three ovarian tumors. *Cancer Genet. Cytogenet.*, **26**, 327–337.

LAMMIE, G.A. & PETERS, G. (1991). Chromosome 11q13 abnormalities in human cancer. *Cancer Cells*, **3**, 414–420.

LARSSON, C., SKOGSEID, B., ÖBERG, K., NAKAMURA, Y. & NORDENSKJÖLD, N. (1988). Multiple endocrine neoplasia type 1 gene maps to chromosome 11 and is lost in insulinoma. *Nature*, **332**, 85–87.

LEE, J.H., KAVANAGH, J.J., WHARTON, J.T., WILDRICK, D.M. & BLICK, M. (1989). Allele loss at the c-Ha-ras1 locus in human ovarian cancer. *Cancer Res.*, **49**, 1220–1222.

LEE, J.H., KAVANAGH, J.J., WILDRICK, D.M., WHARTON, J.T. & BLICK, M. (1990). Frequent loss of heterozygosity on chromosomes 6q, 11 and 17 in human ovarian carcinomas. *Cancer Res.*, **50**, 2724–2728.

LI, S.-B., SCHWARTZ, P.E., LEE, W.H. & YANG-FENG, T.L. (1991). Allele loss at the retinoblastoma locus in human ovarian cancer. *J. Natl Cancer Inst.*, **83**, 637–640.

MIOTTI, S., CANEVARI, S., MENARD, S., MEZZANZANICA, D., PORRO, G., PUPA, S.M., REGANZZONI, M., TAGLIABUE, E. & COLNAGHI, M.I. (1987). Characterisation of human ovarian carcinoma-associated antigens defined by novel monoclonal antibodies with tumor-restricted specificity. *Int. J. Cancer*, **39**, 297–303.

MOTOKURA, T., BLOOM, T., KIM, H.G., JUPPNER, H., RUDERMAN, J.V., KRONENBERG, H.M. & ARNOLD, A. (1991). A novel cyclin encoded by a *bcl1*-linked candidate oncogene. *Nature*, **350**, 512–515.

MÜLLENBACH, R., LAGODA, P.J.L. & WELTER, C. (1989). An efficient salt-chloroform extraction of DNA from blood and tissues. *Trends Genet.*, **5**, 391.

PEJOVIC, T., HEIM, S., MANDHAL, N., ELMFORS, B., FLODERUS, U.-M., FURGYIK, S., HELM, G., WILLEN, H. & MITELMAN, F. (1989). Consistent occurrence of a 19p+ marker chromosome and loss of 11p material in ovarian seropapillary cystadenocarcinomas. *Genes Chromosom. Cancer*, **1**, 167–171.

PEJOVIC, T., HEM, S., MANDHAL, N., ELMFORS, B., FLODERUS, U.-M., FURGYIK, S., HELM, G., WILLEN, H. & MITELMAN, F. (1990). Trisomy 12 is a consistent chromosomal aberration in benign ovarian tumors. *Genes Chromosom. Cancer*, **2**, 48–52.

PEJOVIC, T., HEIM, S., MANDHAL, N., BALDETORP, B., ELMFORS, B., FLODERUS, U.-M., FURGYIK, S., HELM, G., HIMMELMANN, A., WILLEN, H. & MITELMAN, F. (1992). Chromosome aberration in 35 primary ovarian carcinomas. *Genes Chromosom. Cancer*, **4**, 58–68.

RAGOUSSIS, J., SENGER, G., TROWSDALE, J. & CAMPBELL, I. (1992). Genomic organisation of the human folate receptor genes on chromosome 11q13. *Genomics*, **14**, 423–430.

RATNAM, M., MARQUARDT, H., DUHRING, J.L. & FREISHEIM, J.H. (1989). Homologous membrane folate binding proteins in human placenta: cloning and sequence of a cDNA. *Biochemistry*, **28**, 8249–8254.

ROSENBERG, C.L., KIM, H.G., SHOWS, T.B., KRONENBERG, H.M. & ARNOLD, A. (1991). Rearrangement and overexpression of D11S-287E, a candidate oncogene on chromosome 11q13 in benign parathyroid tumours. *Oncogene*, **6**, 449–453.

RUSSELL, P. (1987). Common epithelial tumours of the ovary In: *Obstetrical and Gynaecological Pathology*, Third edition, Vol. 1, Fox, H. (ed.). pp. 556–622. Churchill Livingstone: Edinburgh.

RUSSELL, S.E.H., HICKEY, G.I., LOWRY, W.S., WHITE, P. & ATKINSON, R.J. (1990). Allele loss from chromosome 17 in ovarian cancer. *Oncogene*, **5**, 1581–1583.

SASANO, H., GARRETT, C.T., WILKINSON, D.S., SILVERBERG, S., COMERFORD, J. & HYDE, J. (1990). Protooncogene amplification and tumor ploidy in human ovarian neoplasms. *Hum. Pathol.*, **21**, 382–391.

SATO, T., SAITO, H., MORITA, R., KOI, S., LEE, J.H. & NAKAMURA, Y. (1991). Allelotype of human ovarian cancer. *Cancer Res.*, **51**, 5118–5122.

SCHUURING, E., VERHOEVEN, E., MOOI, W.J. & MICHALIDES, R.J.A.M. (1992). Identification and cloning of two overexpressed genes, U21B31/*PRAD*1 and *EMS*1, within the amplified chromosome 11q13 region in human carcinomas. *Oncogene*, **7**, 355–361.

SEROV, S.F., SCULLY, R.E. & SOBRIN, L.H. (1973). Histological typing of ovarian tumours. In: *International Histological Classification of Tumours*, Number 9. pp. 17–54. World Health Organization: Geneva.

SRIVATSAN, E.S., MISRA, B.C., VENUGOPALAN, M. & WILCZYNSKI, S.P. (1991*a*). Loss of heterozygosity for alleles on chromosome 11 in cervical carcinoma. *Am. J. Hum. Genet.*, **49**, 868–77.

SRIVATSAN, E.S., MURALI, V. & SEEGER, R.C. (1991*b*). Loss of heterozygosity for alleles on chromosome 11q and 14q in neuroblastoma. *Prog. Clin. Biol. Res.*, **366**, 91–98.

TANIGAMI, A., TOKINO, T., TAKIGUCHI, S., MORI, M., GLASER, T., PARK, J.W., JONES, C. & NAKAMURA, Y. (1992). Mapping of 262 DNA markers into 24 intervals on human chromosome 11. *Am. J. Hum. Genet.*, **50**, 56–64.

THAKKER, R.V., BOULOUX, P., WOODING, C., CHOTAI, K., BROAD, P.M., SPURR, N.K., BESSER, G.M. & O'RIORDAN, J.L.H. (1989). Association of parathyroid tumors in multiple endocrine neoplasia type 1 with loss of alleles on chromosome 11. *N. Engl. J. Med.*, **321**, 218–224.

TSAO, S.W., MOK, C.-H., OIKE, K., MUTO, M., GOODMAN, H.M., SHEETS, E.E., BERKOWITZ, R.S., KNAPP, R.C. & LAU, C.C. (1991). Involvement of p53 gene in the allele deletion of chromosome 17p in human ovarian tumours. *Anticancer Res.*, **11**, 1975–1982.

TSUJIMOTO, Y., JAFFE, E., COSMAN, J., GORMAN, J., NOWELL, P.C. & CROCE, C.M. (1985). Clustering of breakpoints on chromosome 11 in human B-cell neoplasms with the t(11;14) chromosome translocation. *Nature*, **315**, 340–343.

VIEL, A., DE PASCALE, L., TOFFOLI, G., TUMIOTTO, L., MIOTTO, E. & BIOCCHI, M. (1991). Frequent occurrence of H-ras1 alleleic deletion in human ovarian adenocarcinomas. *Tumori*, **77**, 16–20.

WARNICH, L., GROENEWALD, I., THEART, L. & RETIEF, A.E. (1992). Highly informative dinucleotide repeat polymorphism at the D11S29 locus on chromosome 11q23. *Hum. Genet.*, **89**, 357–359.

WEBER, J.L., KWITEK, A.E. & MAY, P.E. (1990). Dinucleotide repeat polymorphisms at the D11S419 and *CD3D* loci. *Nucleic. Acids Res.*, **18**, 4036.

ZIEMAN-VAN DER POEL, S., MCCABE, N.R., GILL, H.J., ESPINOSA, R., PATEL, Y., HARDEN, A., RUBINELLI, P., SMITH, S.D., LEBEAU, M.M., ROWLEY, J.D. & DIAZ, M.O. (1991). Identification of a gene, *MLL*, that spans the breakpoint in 11q23 translocations associated with human leukemias. *Proc. Natl Acad. Sci. (USA)*, **88**, 10735–10739.